# Differences in virulence between the two more prevalent *Staphylococcus aureus* clonal complexes in rabbitries (CC121 and CC96) using an experimental model of mammary gland infection

**DOI:** 10.1186/s13567-020-0740-1

**Published:** 2020-02-13

**Authors:** Mariola Penadés, David Viana, Ana García-Quirós, Asunción Muñoz-Silvestre, Elena Moreno-Grua, Sara Pérez-Fuentes, Juan José Pascual, Juan M. Corpa, Laura Selva

**Affiliations:** 1 Biomedical Research Institute (PASAPTA-Pathology Group), Facultad de Veterinaria, Universidad Cardenal Herrera-CEU, CEU Universities. C/Tirant lo Blanc 7, Alfara del Patriarca, 46115 Valencia, Spain; 2 Institute for Animal Science and Technology, Universitat Politècnica de València, Camino de Vera 14, 46071 Valencia, Spain

## Abstract

Staphylococcal mastitis is a major health problem in humans and livestock that leads to economic loss running in millions. This process is currently one of the main reasons for culling adult rabbit does. Surprisingly, the two most prevalent *S. aureus* lineages isolated from non-differentiable natural clinical mastitis in rabbits (ST121 and ST96) generate different immune responses. This study aimed to genetically compare both types of strains to search for possible dissimilarities to explain differences in immune response, and to check whether they showed similar virulence in in vitro tests as in experimental intramammary in vivo infection. The main differences were observed in the enterotoxin gene cluster (egc) and the immune-evasion-cluster (IEC) genes. While isolate ST121 harboured all six egc cluster members (*seg*, *sei*, *selm*, *seln*, *selo*, *selu*), isolate ST96 lacked the egc cluster. Strain ST96 carried a phage integrase Sa3 (Sa3int), compatible with a phage integrated into the *hlb* gene (β-haemolysin-converting bacteriophages) with IEC type F, while isolate ST121 lacked IEC genes and the *hlb* gene was intact. Moreover, the in vitro tests confirmed a different virulence capacity between strains as ST121 showed greater cytotoxicity for erythrocytes, polymorphonuclear leukocytes and macrophages than strain ST96. Differences were also found 7 days after experimental intramammary infection with 100 colony-forming units. The animals inoculated with strain ST121 developed more severe gross and histological mastitis, higher counts of macrophages in tissue and of all the cell populations in peripheral blood, and a significantly larger total number of bacteria than those infected by strain ST96.

## Introduction

Mastitis is a very common disease with loss running in millions in the dairy industry worldwide [1, 2]. Mastitis aetiology varies, but *Staphylococcus aureus* stands out in the Gram-positive bacteria that causes this disease [2, 3]. Rabbits suffer natural staphylococcal infections, and clinical mastitis is one of the main problems why commercial rabbit farms close down due to acute and chronic outbreaks [4–6].

Regarding the main strains involved in *S. aureus* infections, subpopulations of *S. aureus* Clonal Complex 121 (multi-locus sequence typing-defined clones ST121) are associated with distinct clinical entities in humans [7], and are a particular common cause of human skin and soft tissue infections [8]. In the last 30–40 years, rabbit farming intensification in the developed world has coincided with a highly virulent epidemic clone of *S. aureus* emerging, also known as ST121, which is associated with the most chronic staphylococcal rabbit infections, mainly skin abscesses, pododermatitis and mastitis on commercial rabbitries [9, 10]. However, other less common lineages can be involved in staphylococcal rabbit infections, such as ST96, which is the second commonest lineage defined by multi-locus sequence typing (MLST) involved in rabbit staphylococcal mastitis [11]. The characterisation of the immune response in natural clinical mastitis cases in rabbits caused by *S. aureus* has shown that animals infected by ST96 strains develop a different immune response than those infected by ST121 strains [11].

*S. aureus* possesses a wide range of virulence factors that play an important role during host infection, such as surface proteins responsible for adhesion and invasion of host cells, exoproteins responsible for immune evasion mechanism, and a number of pore-forming and haemolytic toxins [12]. Adhesion of *S. aureus* to the epithelium of mammary glands plays a crucial role in mastitis developing and, to a great extent, the virulence of strains in mastitis depends on the production of several toxins [13–15]. Staphylococcal virulence factors have been identified in many *S. aureus* collections isolated from intramammary infection cases. However, the factors specifically associated with mastitis remain unknown given the redundancy, diversity, and wide variations in the presence of these genes among *S. aureus* isolates.

Animal models of mastitis are very valuable for studying the pathogenesis of staphylococcosis. The most frequently used animal species in experimental models have been cows [16, 17], goats [18], rabbits [19, 20], rats [21] and mice [22]. However, performing successful experimental infections in species that are not natural hosts for *S. aureus*, such as mice [23], require the genetic adaptation of bacteria prior to inoculation [24], or using very high bacterial doses (10^7^ to 10^9^ colony-forming units [CFU]). Therefore, the rabbit, as a natural host of this pathogen, is a more convenient experimental model to be used to resemble natural infection conditions.

Several studies in the literature report experimental infections by *S. aureus* in rabbit. However, rabbit has been normally used as a model to either study staphylococcal infections in other species [20, 25] or focus on the pathogenesis of staphylococcal skin infections [26, 27].

As both ST121 and ST96 *S. aureus* strains cause severe clinical mastitis, but generate different immune responses in rabbit does under field conditions, the aim of this work was to know whether there were any genetic differences to justify these different responses and to confirm this bacterial behaviour by in vitro and in vivo tests by developing, to this end, a novel experimental model at low-infection doses. Therefore, the specific work aims were to: (1) study the virulence genes involved in the production of adhesins and main toxins in strains ST121 and ST96; (2) analyse the virulence of strains ST121 and ST96 by in vitro tests, as well as their infection capacity in mammary glands using a low-dose in vivo experimental model in which local and peripheral immune responses were compared.

## Materials and methods

### Bacterial strains

This study focused on two representative rabbit *S. aureus* strains, which were selected from a large collection based on their respective MLST. They were isolated from rabbitries with chronic staphylococcosis problems. Isolates ST121 and ST96 were selected as they represent the most prevalent clones in chronic staphylococcal mastitis in rabbits [11]. Strains Jwt (ST121) [28] and DL9 (ST96) were used for experimental mammary infection and the (polymerase chain reaction) PCR analysis of bacterial virulence genes. For the in vitro tests (haemolytic and lysis assays), three representative strains of each ST were compared: ST121 (Jwt, CEU268, CEU890) and ST96 (DL9, CEU852, CEU886). Detailed information on all *S. aureus* strains used is provided in Table 1.

**Table 1:** **Characteristics of the rabbit*Staphylococcus aureus*strains used**

Strain	ST^a^	Geografic origin	Isolation date	Reference
Jwt	121	France	2002	[28]
CEU268	121	Spain	2007	This study
CEU890	121	Spain	2016	This study
DL9	96	Spain	2003	This study
CEU852	96	Spain	2016	This study
CEU886	96	Spain	2016	This study

^a^ST: sequence type defined by multilocus sequence typing (MLST).

### PCR analysis of bacterial virulence genes

Fifty determinants were examined for the presence of the gene by PCR as previously described [29]. The sequences of the oligonucleotide primers, thermocycler programmes and references are summarised in Additional file 1. Each amplification comprised 100 ng of the DNA template, 100 pmol of each primer, 200 μM of (each) deoxynucleoside triphosphate (dATP, dGTP, dCTP, and dTTP), 1× buffer (Netzyme^®^, Epica SL, Valencia, Spain), 1 mM MgCl2, and 1 U of thermostable DNA polymerase (Netzyme^®^, Epica SL). Water was added to a final volume of 25 μL and thermal cycling was performed. The size of the PCR products was determined by electrophoresis on 1% (wt/vol) agarose gels.

### In vitro tests

#### Haemolytic assays

Haemolytic assays were carried out as previously described [30]. Briefly, whole blood was washed twice with phosphate-buffered saline (PBS) and resuspended in PBS to obtain 8% (wt/vol.). Blood was used within the first hour. To prepare the supernatants, five isolated colonies of each strain (ST121: Jwt, CEU268, CEU890 and ST96: DL9, CEU852, CEU886) were sown in 5 mL of TSB and kept at 37 °C for 6 h with shaking. All the cultures were centrifuged for 10 min at 3000 RPM and the supernatant was filtered. Then 100 μL of 8% red blood cells (RBC) were mixed with 100 μL of the different dilutions of the culture supernatants (100%, 50%, 25% and 10%) in 1.5 mL tubes, which resulted in 4% of the final RBC. The mixture was incubated at 37 °C for 30 min and centrifuged for 2 min at 7 K RPM in a minispin centrifuge (Eppendorf, Hamburg, Germany). Next 150 μL of supernatants were transferred to ELISA plates (Sigma-Aldrich, Missouri, USA) without disturbing the pellet. OD 450 nm was read to determine the haemolysis capacity of strains ST121 and ST96. For the positive control, RBC were mixed with 20% Triton X-100. For negative control, RBC were mixed with PBS. This protocol was carried out using three strains of each ST type to compare the virulence between strains ST121 and ST96.

#### Polymorphonuclear leukocytes and macrophage lysis assays

The lactate dehydrogenase (LDH) release assay was performed for the high-throughput quantification of cell death and cell lysis. Briefly, 10 μL of the different dilutions of culture-filtered supernatants (similar to those in the haemolytic assays) were added to 2 × 10^6^ neutrophils/mL or 3 × 10^5^ macrophages/mL up to a total volume of 100 μL to be incubated for 30 min at 37 °C. Supernatants were then collected and the release of LDH was measured following the manufacturer’s recommendations (Cytotoxicity Detection Kit^PLUS^, Roche, Basel, Switzerland). To determine the spontaneous LDH and maximum LDH releases, the controls indicated by the manufacturer were used. Polymorphonuclear leukocytes (PMN) and macrophage purification were carried out according to previously described protocols [31, 32]. Briefly, rabbit blood was collected from the central artery of ear in a conical 50 mL tube with acid citrate dextrose at the 4:1 ratio (v/v). To the PMN purification, Hetastarch (Sigma-Aldrich) was added to cause sedimentation of erythrocytes. White blood cells were resuspended in rabbit neutrophil buffer (138 mM NaCl, 27 mM KCl, 8.1 mM Na_2_HPO_4_, 1.5 mM KH_2_PO_4_, and 5.5 mM glucose dissolved in sterile H_2_O and sterile-filtered) and neutrophils were isolated with the gradient by Histopaque 1077 (Sigma-Aldrich). To the monocytes purification, peripheral blood mononuclear cells (PBMCs), including monocytes, were isolated using Lymphoprep (Palex, Madrid, Spain). After the density gradient (800 g for 20 min in the non-accelerator and non-brake modes), the isolated PBMCs were washed twice with PBS. Cells were resuspended in RPMI-1640 medium with 10% foetal bovine serum (FBS) and 1% penicillin/streptomycin (ThermoFisher, Massachusetts, USA). Subsequently, cell suspensions were transferred to 6-well flat-bottom tissue culture plates (Sigma). After incubation for 2 h at 37 °C in humidified 5% CO_2_ in a gas incubator, the non-adherent cells were removed by washing with PBS. To generate the in vitro polarisation of macrophages, the adherent cells were cultured for 6 days in RPMI-1640 medium with 10% FBS and 1% penicillin/streptomycin supplemented with recombinant human granulocyte/macrophage colony-stimulating factor (GM-CSF, Gibco, ThermoFisher). To quantify both cell number and viability, a haemocytometer and trypan blue were used. Following the same criteria and aims as for the haemolysis protocol, these assays were performed using three strains from each ST herein included (ST121: Jwt, CEU268, CEU890 and ST96: DL9, CEU852, CEU886).

### Experimental mammary infection

#### Preparing the inoculum for infection

Strains ST121 and ST96 were cultured overnight from frozen stocks in trypticase soya agar (TSA). The next morning, 5 CFU were selected randomly and cultured for 6 h in trypticase soya broth (TSB) at 37 °C with shaking (240 rpm). To prepare inocula, bacteria were washed in Dulbecco’s phosphate-buffered saline (DPBS) and resuspended in DPBS at 1000 CFU/mL, as previously described [28]. Bacterial preparations were used immediately for the inoculation of rabbits, as described below.

#### Animal and experimental procedures

Twenty-four pregnant primiparous albino hybrid rabbit does (*Oryctolagus cuniculus*), aged 22–26 weeks and weighing 2.0–2.5 kg, were housed individually in wire mesh cages. They received commercial feed and water ad libitum. Bacterial inoculation was performed 2 days post-partum (dpp) by keeping kits with their mothers. The mammary gland skin was shaved and, together with teat tips, was aseptically cleaned and thoroughly disinfected before inoculation using diluted chlorhexidine spread over tissue using sterile gauzes. Prior to injection, 100 µL of milk samples were taken using sterile swabs (Sarstedt, Nümbrecht, Germany). They were cultured in blood agar plates to check the presence of bacteria, which would have been an exclusion criterion. None of them tested positive. Rabbits were randomly separated into three groups and received a single intramammary injection into the lactiferous nipple of the second left mammary gland as follows: (i) control group (*n* = 6) received 0.1 mL DPBS; (ii) the ST96 group (*n* = 9) received 100 CFU in 0.1 mL DPBS; (iii) the ST121 group (*n* = 9) received 100 CFU in 0.1 mL DPBS. Animals’ general health status and gross lesion characteristics were recorded daily for 7 days. Abscess dimensions were measured by a Vernier caliper. Length (L) and width (W) were used to calculate the abscess area (A = Π[L × W]/2) [33]. Macroscopic lesions were evaluated by their abscess area as follows: Healthy (no apparent lesions); Mild (< 10 cm^2^); Moderate (10–50 cm^2^); Severe (> 50 cm^2^ or with gross necrosis). Body temperatures and blood samples were collected on 0, 1, 3 and 7 days post-inoculation (dpi). Animals were euthanised after 7 days by intravenous barbiture injection (Dolethal, Vétoquinol, Madrid, Spain). To determine CFU, tissue samples were weighed, homogenised in DPBS, diluted in saline and plated on TSA. CFU were counted the next day.

The study protocol was approved by the Animal Ethics and Welfare Committee of the CEU Cardenal Herrera University, Valencia, Spain (Approval number: 11/003; date of approval: January 2011).

#### Haematology and flow cytometric analyses

Blood samples (1 mL) were collected in EDTA vacuum tubes from the median artery of the ear. White blood cell (WBC) counts and lymphocyte proportions were determined in a haematology analyser (MEK-6410, Nihon Kohden, Tokyo, Japan). The flow cytometric analysis of white blood cells was performed using specific primary antibodies and secondary antibodies (see [11]), as previously described [34, 35]. The relative and absolute numbers of lymphocytes and lymphocyte subpopulations were calculated as described by Hulstaert et al. [36].

#### Histological studies

Mammary glands were removed from all the animals while a complete necropsy was performed. Tissues were fixed in 10% neutral buffered formalin (for 24–36 h) and dehydrated through graded alcohols before being embedded in paraffin wax. Several 4 μm-thick sections were cut from each sample and stained by haematoxylin and eosin (HE). All the sections were evaluated under a light microscope (Leica MC190HD, Wetzlar, Germany).

The microscopical lesions in mammary glands were classified according to histomorphological characteristics following the criteria established by Viana et al. [10]. Hence, four different types of mastitis were defined: abscessation, suppurative mastitis with lobular pattern, cellulitis and mixed lesions. Briefly, abscessation was characterised by one abscess or by several of variable sizes. Depending on the degree of the demarcation of abscesses, according to the presence and composition of their capsule, they were classified as compact, non-compact and non-encapsulated abscesses. Suppurative mastitis with a lobular pattern consisted of non-encapsulated areas with suppurative inflammation where bacterial colonies were present and affected mammary lobules, which caused the necrosis of the epithelial cells of the mammary alveoli, and triggered the presence of numerous heterophils and occasional macrophages in alveolar lumina. Cellulitis was characterised by a broad band of inflammatory tissue surrounding the mammary gland, composed of heterophils, macrophages, and sometimes plasma cells, which affected subcutaneous tissue, and even the abdominal musculature. Mixed-type mastitis presented common characteristics to two of the above-described histological features: abscessation and cellulitis.

#### Immunohistochemical studies

Immunohistochemical staining was undertaken for T-lymphocytes (monoclonal anti-canine CD3, clone CD3-12, UC Davis, USA), B-lymphocytes (monoclonal mouse anti-human CD79αcy, clone HM57, Dako, California, USA), macrophages (monoclonal mouse anti-rabbit Ab-5, clone RAM11, Dako) and plasma cells (polyclonal goat anti rabbit-IgG:HRP conjugate, Stressgen, Enzo Biochem, New York, USA) by the avidin–biotin–peroxidase complex (ABC) method at the dilutions recommended by the manufacturer. Positive cells were enumerated in 20 randomly selected fields (400×) bordering the area of necrosis. This represented a total area of 1.6 mm^2^ per slide.

### Statistical analysis

The variables addressed to evaluate the in vitro lysis capacity of the supernatants from the different strains were analysed using a split-plot model as measurements have an equal variance at all the times and dilutions, and pairs of measurements from the same strain were equality correlated (the Huynh–Feldt condition [37]). The model included the ST classification (ST96 and ST121), the dilution of supernatants (10%, 25%, 50% and 100%), repetition day (1, 2 and 3) and their interactions as fixed effects, as well as the strain (ST) as a random effect.

The data from the flow cytometry and immunohistochemistry studies were analysed using a general linear model (SAS/STAT version 9.2, SAS Institute, North Carolina, USA). The flow cytometry data did not demonstrate normal distribution, and log_10_ transformation was applied prior to the analysis. The statistical model included only lesion type as the fixed effect. The least square means comparison was made by a Student’s *t* test. The effect of the control level of the abscess (compact, loose, non-encapsulated) and the spreading level of the lesion (unifocal, multifocal, lobular pattern) on the immunohistochemistry parameters were determined using contrasts as the difference between their various levels (by testing their significant difference from 0; *P* < 0.05). The effect of the control level of the abscess, the spreading level of the lesion and the MLST (levels: 96, 121) on lymphocyte populations was determined using contrasts as the rate between their different levels (by testing their significant difference from 1; *P* < 0.05). To test the relation between the immunohistochemistry parameters in lesions and the lymphocyte populations detected by flow cytometry, Pearson’s correlation coefficients (*ρ*) were calculated.

## Results

### Bacterial virulence genes

Fifty bacterial virulence genes in *S. aureus* were tested by PCR to analyse their presence in strains ST121 and ST96. Twenty-one virulence determinants were positive in both strains: 13 genes related to host adhesion and invasion (*clfA*, *clfB*, *cna*, *ebpS*, *eno*, *fib*, *fmtB*, *fnbA*, *fnbB*, *icaA*, *map/eap*, *sdrC*, *sdrD*) and eight genes with potential cytotoxic activity (*atlA*, *hla*, *hld*, *hlgC*, *lukAB*, *lukED*, *psmA*, *psmB*). Conversely, it was not possible to detect 17 virulence factors, as identified in other isolates from mastitis, using the oligonucleotide primers available: a catabolic mobile gene (*arcA*), adhesin (*srdE*) and 15 cytotoxins (*lukSF*-PVL, *lukMF*-PV, *sea*, *seb*, *sec*, *sed*, *see*, *seh*, *sej*, *selk*, *sel*, *selq*, *eta*, *etb*, *tst*). Differences were identified between strains ST121 and ST96. Isolate ST121 harboured all six egc cluster members (*seg*, *sei*, *selm*, *seln*, *selo*, *selu*), but isolate ST96 lacked the egc cluster. Strain ST96 harboured *sak*, *scn*, *chp* and *selp* and phage integrase Sa3, and was negative to *hlb*. Finally, Bbp adhesin was only detected in strain ST121.

### In vitro assays

**Figure 1:**
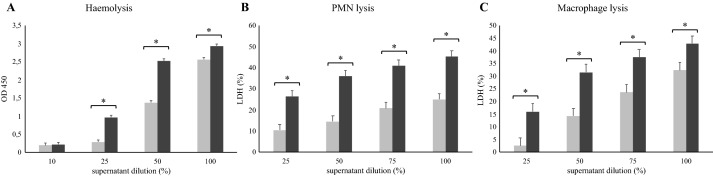
**Comparison of the bacterial virulence corresponding to strains ST121 and ST96 against different in vitro cell populations: erythrocytes, PMN and macrophages. A** Haemolytic assays towards 8% rabbit RBC. OD 450 nm was read to determine the haemolytic capacity of the supernatant from strains ST121 and ST96. **B**, **C** PMN lysis assays and macrophage lysis assays. The supernatants of strains ST121 and ST96 were added to 2 × 10^6^ neutrophils/mL or 3 × 10^5^ macrophages/mL, respectively, and incubated for 30 min at 37 °C. Later the LDH release assay was performed for the quantification of cell death and cell lysis. *OD* optical density, *LDH*: lactate dehydrogenase, *PMN* polymorphonuclear leukocytes, *RBC* red blood cells. ■ Average results of three ST96 strains and ■ Average results of three ST121 strains. The experiments were performed in triplicate. Bars represent the obtained least square means and error bars show their corresponding standard error. *Statistically significant differences (*P* < 0.05).

In order to ensure that the differences in bacterial virulence were not specific of the strains herein included, but of the strains belonging to the same sequence type, Figure 1 shows the cytotoxicity results obtained from using three different strains from ST121 (Jwt, CEU268, CEU890) and ST96 (DL9, CEU852, CEU886). After growing to the mid-exponential phase, the supernatant of all the tested strains ST121 showed greater cytotoxicity activity for erythrocytes (undiluted, dilution, 1/2 and 1/4, *P *< 0.05), PMN and macrophages (all dilutions, *P* < 0.05) than for strains ST96. This finding confirms common behaviour among the strains of the same ST.

### Experimental model

The first result worth mentioning after the experimental infection was that the inoculation of only 100 CFU was able to successfully develop mastitis, a process characterised by the changes observed in the number of cell populations in both peripheral blood and mammary tissue.

#### Temperature evolution

**Figure 2:**
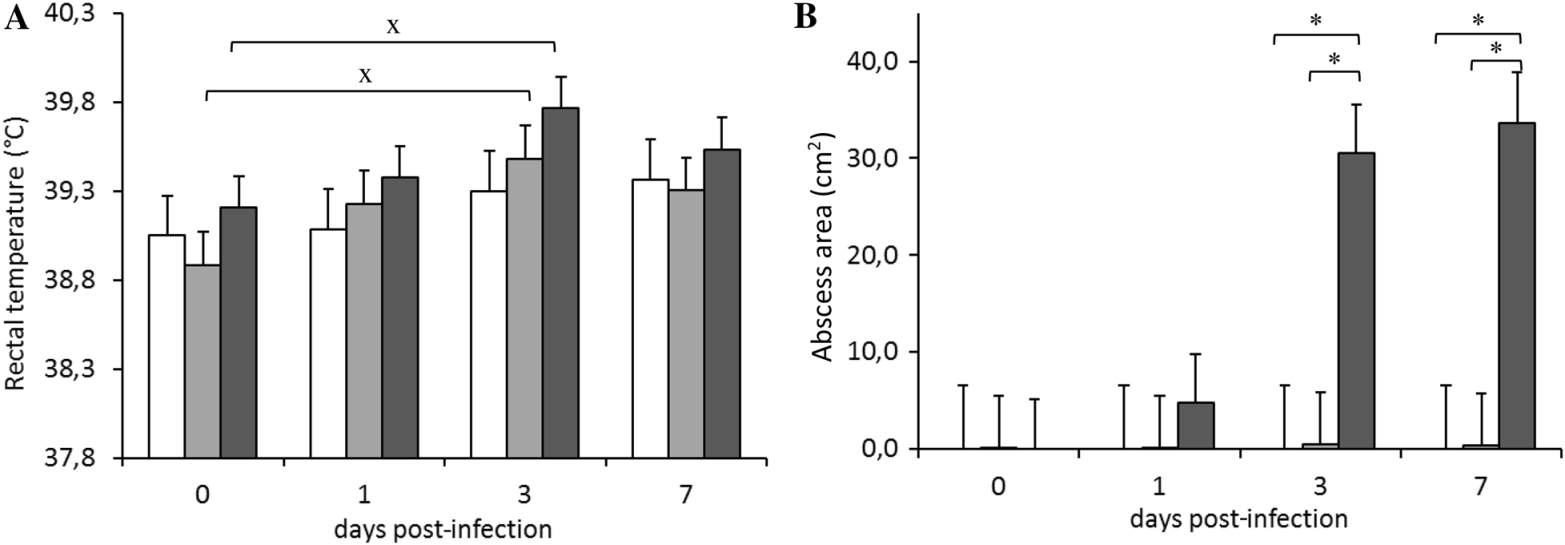
**Evolution of A rectal temperature and B abscess area** (**cm**^**2**^) **throughout the 7-day infection challenge according to the inoculated strain: □ control,** ■ **ST96 and** ■ **ST121.** Bars represent the obtained least square means and error bars show their corresponding standard error. ^x^ Significantly different (*P* < 0.05) to the value before infection (day 0). *Significantly different on each day post-infection (*P* < 0.05).

As rectal temperature evolved, a significant difference in the value before infection (*P* < 0.05) was observed in groups ST96 and ST121 on day 3 (Figure 2A). However, only three individuals from group ST121 had fever on 3 dpi (above 41 °C, with a maximum temperature of 41.5 °C). High temperature was not registered in any of the rabbits infected by strain ST96 or in the control group.

#### Macro- and microscopic lesions

The grossly infected mammary glands showed a thickening or induration of the mammary tissue around or near the teat. Macroscopic lesions were evaluated by abscess area (Figure 2B). The rabbits infected by strain ST121 showed lesions on 1 dpi. On 3 dpi, significant differences appeared in the group infected by strain ST121 compared to 0 dpi, and between groups (ST121 vs. ST96, and ST121 vs. the control group; *P* < 0.05). These differences remained on 7 dpi (Figure 2B, *P* < 0.05).

**Figure 3:**
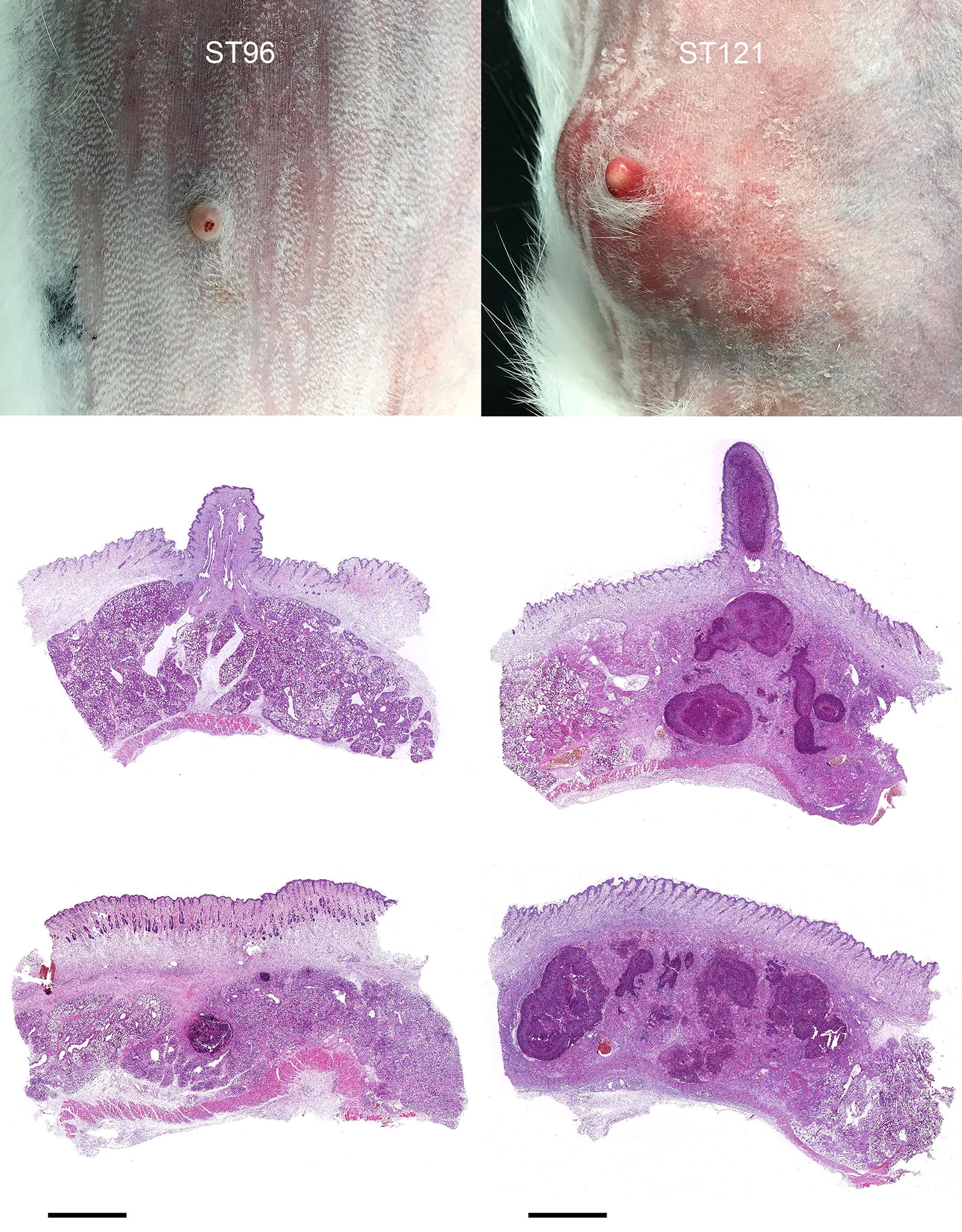
**Comparison of the macroscopic and microscopic lesions in the rabbits from groups ST96** (**left**) **and ST121** (**right**). ST96: lesion classified as mild, characterised by small and scarcely extensive abscesses, some of which were only histologically visible. ST121: lesion classified as moderate, characterised by great mammary gland tissue thickening and induration, produced by several coalescing abscesses. Haematoxylin and Eosin. Bar = 0.2 cm.

Only two rabbits (22.2%) infected by strain ST96 presented macroscopic lesions, whose severity was remarkably less marked than that observed in the rabbits from group ST121. They were classified as mild. Seven rabbits infected by strain ST121 showed macroscopic lesions: three were moderate and four were severe (Table 2, Figure 3). Histopathology revealed inflammatory lesions in four and eight rabbits infected by strain ST96 and strain ST121, respectively (Table 3). After examining the classification of the four main types described in natural mastitis according to their histological characteristics [10], the lesions caused by strain ST121 were classified as: four compact-capsule multifocal abscesses, two suppurative mastitis with a lobular pattern, one cellulitis and one mixed. The lesions caused by strain ST96 were classified as: one compact-capsule abscess unifocal and three suppurative lesions with a lobular pattern.

**Table 2:** **Number of animals showing each lesion type (according to the severity criteria established for macroscopic appearance) produced in the mammary glands of the rabbit does from all the experimental groups at the end of experimental infection (7** **days post-infection)**

	Gross lesions		
	ST121 (*n* = 9)	ST96 (*n* = 9)	Control (*n* = 6)
Healthy	2	7	6
Mild	0	2	0
Moderate	3	0	0
Severe	4	0	0

**Table 3:** **Number of animals showing each lesion type (according to the classification based on histological appearance) produced in the mammary glands of the rabbit does from all the experimental groups at the end of experimental infection (day 7 post-infection)**

	Microscopic lesions		
	ST121 (*n* = 9)	ST96 (*n* = 9)	Control (*n* = 6)
Compact-capsule multifocal abscesses	4	0	0
Compact-capsule unifocal abscesses	0	1	0
Suppurative mastitis (lobular pattern)	2	3	0
Cellulitis	1	0	0
Mixed	1	0	0
No lesions	1	5	6

#### Number of bacteria from lesions

The number of *S. aureus* CFU per mammary gland sample on day 7 was also evaluated. No bacteria were recovered from the control group tissues. However, bacteria were recovered from all the animals infected by strain ST96, except for two rabbits that did not develop mastitis. The highest counts were detected in the lesions classified as mild. Bacteria were recovered from all the mammary glands inoculated with ST121. The number of CFU per abscess was significantly bigger for the animals infected by ST121 than by ST96 (an increase of +97% CFU; *P* < 0.05) (Table 4). Moreover, regardless of the inoculated strain, moderate and severe lesions were in the order of 10^6^ and 10^7^ CFU/g, respectively. The animals with mild lesions did not exceed 10^4^ CFU/g and those without lesions did not exceed 10^3^ CFU/g.

**Table 4:** **Bacterial concentration and leukocyte counts performed with the immunohistochemistry slides of the mammary tissue from all the different experimental groups taken at the end of the immunological challenge (day 7 experimental post-infection)**

	Group						
	Control		ST96		ST121		*P*-value
No. of animals	6		9		9		
Total bacteria (log_10_[CFU]/g)	− 2.00	± 0.80^a^	1.04	± 0.65^b^	6.23	± 0.65^c^	0.0001
Leukocytes counts (cells/mm^2^)							
CD3^+^							
At nipple	27.5	± 33.5^a^	97.5	± 27.4^ab^	163.5	± 27.4^b^	0.0176
1 cm from nipple	18.13	± 32.57	59.57	± 26.66	97.51	± 26.66	0.1920
RAM11^+^							
At nipple	84.1	± 61.2^a^	219.9	± 50.1^a^	435.5	± 50.1^b^	0.0007
1 cm from nipple	29.4	± 58.1^a^	99.5	± 47.5^ab^	218.9	± 47.5^b^	0.0519
CD79^+^							
At nipple	82.5	± 38.5	141.2	± 31.5	147.0	± 31.5	0.3967
1 cm from nipple	87.75	± 23.75	91.63	± 19.51	66.43	± 19.51	0.6345
IgG^+^							
At nipple	339.6	± 32.3	293.1	± 26.4	343.8	± 26.4	0.3599
1 cm from nipple	336.3	± 32.5^b^	255.3	± 26.6^ab^	210.0	± 26.6^a^	0.0624

*CFU* colony-forming units.

^abc^Means in a row not sharing superscripts significantly differ at *P* < 0.05.

#### Local immune response

**Figure 4:**
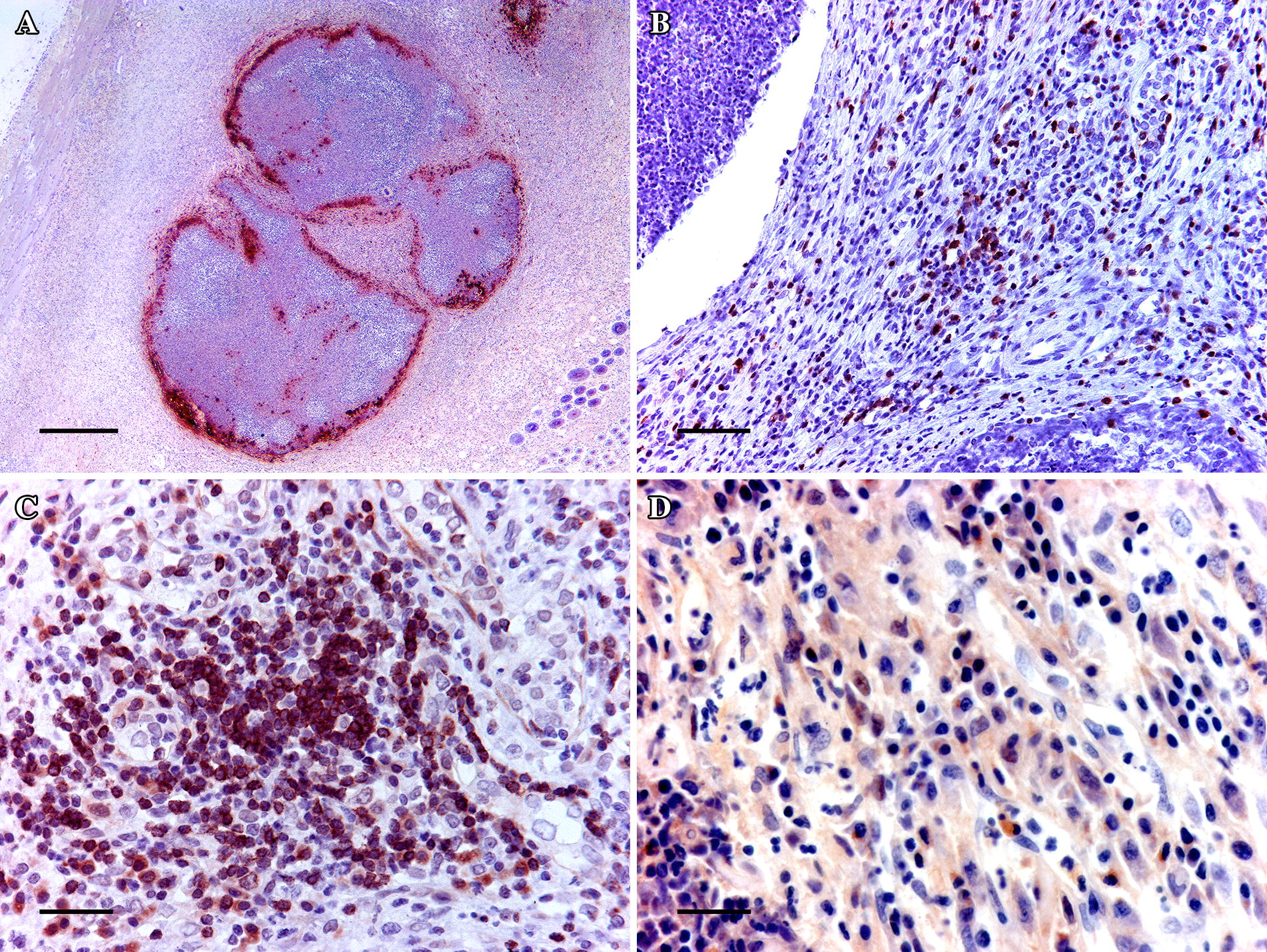
**Mammary gland lesions immunohistochemically stained showing different locations of the marked cells**. **A** Macrophages (RAM11^+^) are located peripherally to the necrotic area to form “palisades”, and to separate necrotic tissue from healthy tissue. Scale bar = 500 µm. **B** An encapsulated abscess surrounded by loose connective tissue reveals the presence of abundant T-lymphocytes (CD3^+^) entangled in the layers of loose connective tissue around the abscess. Scale bar = 50 μm. **C** Proliferation of B-lymphocytes (CD79αcy^+^) in the mammary interstitium arranged in lines and forming strands. Scale bar = 25 μm. **D** Peripheral area of suppurative mastitis with a lobular pattern in which many cells are plasma cells (IgG^+^). Scale bar = 25 μm.

Table 4 presents the leukocyte counts observed on the immunohistochemical slides made on the mammary tissue from all the experimental groups, sampled on 7 dpi. Despite all the positive cells in nipple sections being generally more numerous for ST121 mastitis compared to ST96 mastitis or the control group, the increase was only statistically significant (*P* < 0.05) for the number of macrophages (RAM11^+^). Macrophages (RAM11^+^) were located peripherally to necrotic areas and formed “palisades” by separating necrotic from healthy tissue (Figure 4A). For the encapsulated abscesses, macrophages were present on inner capsule layers. T-lymphocytes (CD3^+^) appeared around the necrotic area, and were even randomly entangled throughout loose connective tissue fibres (Figure 4B). B-lymphocytes (CD79^+^) tended to appear further away from the edge of necrosis to form small strands with one another regardless of abscess type (Figure 4C). Plasma cells (IgG^+^) appeared in the connective tissue adjacent to the necrotic foci in the mammary interstitium (Figure 4D).

In the tissue sections taken 1 cm away from nipples, oscillations in the number of leukocytes were also observed depending on the inoculated strain (Table 4), but none was statistically significant.

#### Peripheral immune response

**Figure 5:**
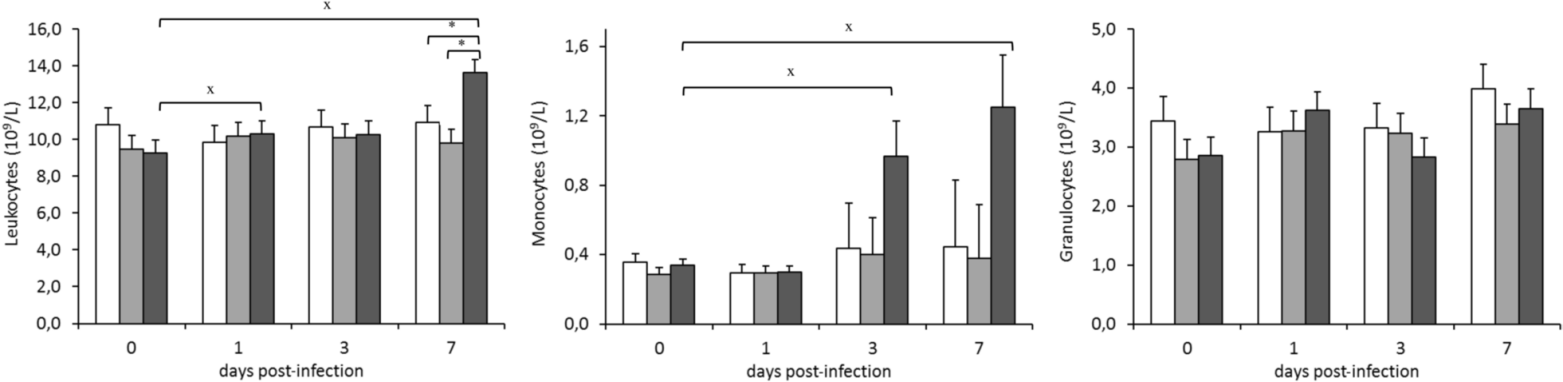
**Evolution of leukocyte, monocyte and granulocyte counts in peripheral blood throughout the 7-day infection challenge according to the inoculated strain: □ control,** ■ **ST96 and** ■ **121.** Bars represent the obtained least square means and error bars show their corresponding standard error. ^x^ Significantly different (*P* < 0.05) to the value before infection (day 0). * Significantly different on each day post-infection (*P* < 0.05).

Figure 5 shows the flow cytometric analysis results of peripheral blood cells. The rabbits infected by strain ST121 had relatively higher circulating leucocytes in blood on 1 and 7 dpi, and were significantly different (*P* < 0.05) to day 0. On day 7 dpi, the proportion of circulating leukocytes was higher in those rabbits infected by strain ST121 than in those inoculated with ST96 or the control groups (*P* < 0.05).

The animals infected by strain ST121 had higher proportions of monocytes on days 3 and 7 versus day 0 (*P* < 0.05). Strain type did not affect the proportion of granulocytes in blood.

**Figure 6:**
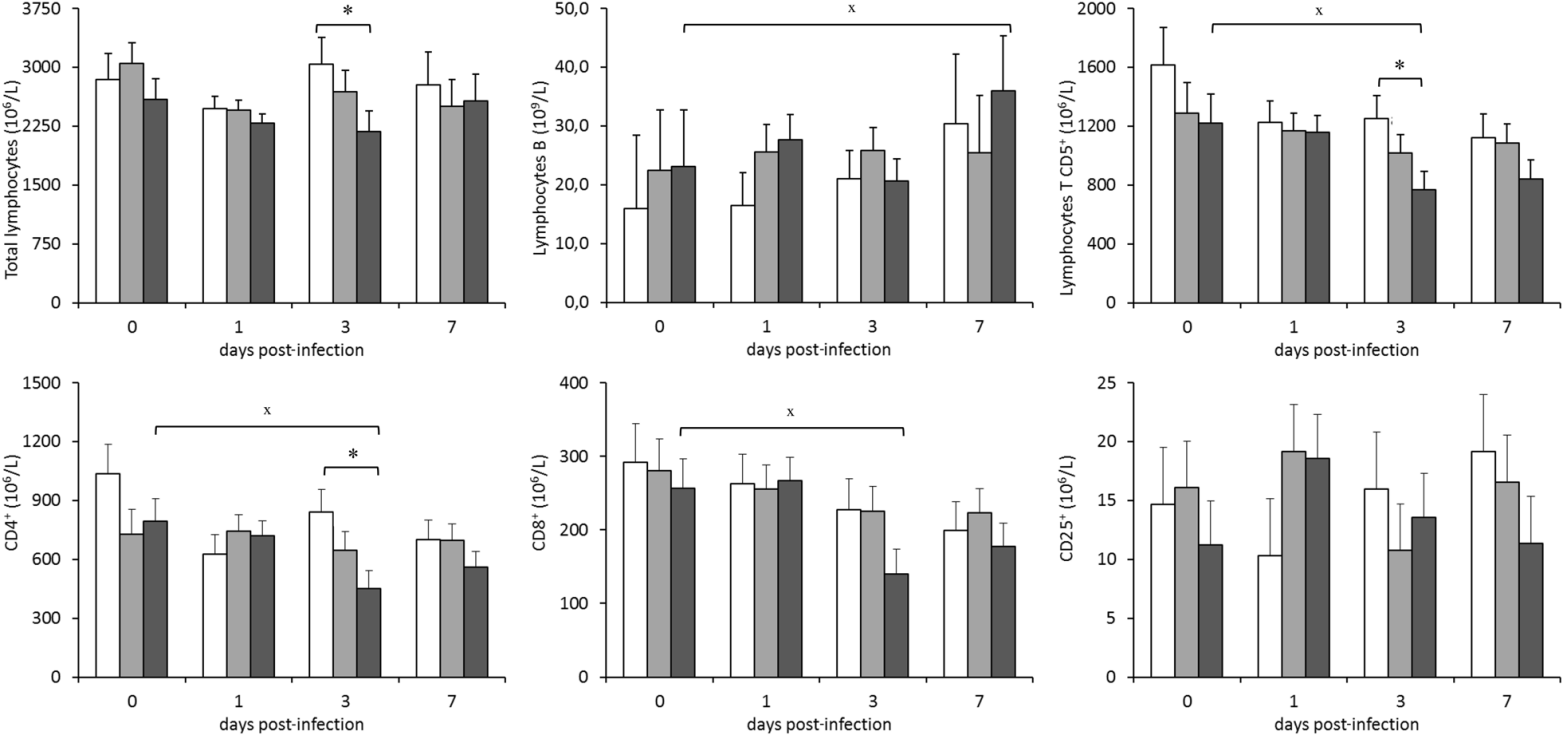
**Evolution of the lymphocyte** (**total, B, T CD5**^**+**^**, CD4**^**+**^**, CD8**^**+**^**and CD25**^**+**^) **counts in peripheral blood throughout the 7-day infection challenge according to the inoculated strain: □ control,** ■ **ST96 and** ■ **ST121.** Bars represent the obtained least square means and error bars show their corresponding standard error. ^x^ Significantly different (*P* < 0.05) to the value before infection (day 0). * Significantly different on each day post-infection (*P* < 0.05).

**Figure 7:**
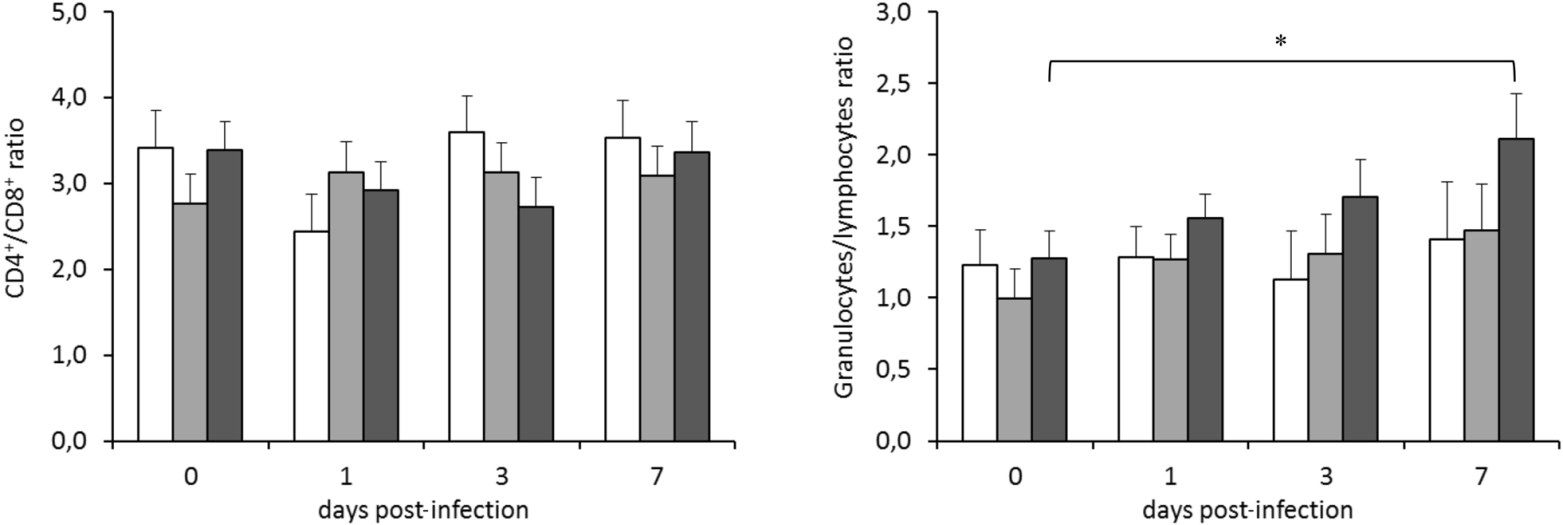
**Evolution of the CD4**^**+**^**/CD8**^**+**^**and granulocytes/lymphocytes ratios in peripheral blood throughout the 7-day infection challenge according to the inoculated strain: □ control,** ■ **ST96 and** ■ **121.** Bars represent the obtained least square means and error bars show their corresponding standard error. * Significantly different (*P* < 0.05) to the value before infection (day 0).

Figure 6 shows the evolution of lymphocyte counts in blood throughout the infection challenge. The rabbits infected by strain ST121 presented the lowest counts of total lymphocytes on 1 and 3 dpi, although differences were only significant compared to the control group on 3 dpi (*P* < 0.05). In the animals infected by strain ST121, the lowest value of the B-lymphocytes was recorded on 3 dpi, before they significantly increased on 7 dpi compared to the value obtained prior to infection (*P* < 0.05). In comparison, B-lymphocytes only showed very slight oscillations throughout infection in the animals inoculated with strain ST96. The most remarkable trait noted for T-lymphocytes (CD5^+^) evolution was the significant decrease (*P* < 0.05) observed in the animals infected by strain ST121 on 3 dpi compared to the initial corresponding counts and control animals, which had still not recovered on 7 dpi (different to B-lymphocytes evolution). This tendency is consistent with the decrease recorded in the subsets of the T-lymphocytes herein included (CD4^+^, CD8^+^ and CD25^+^), but only the first two were significant—*P* < 0.05—, vs. 0 dpi) and were observed simultaneously on 3 dpi in the same group of animals (ST121). Therefore, the evolution of the CD4^+^/CD8^+^ ratio showed no significant variation throughout the experimental infection (Figure 7). Finally, the granulocytes/lymphocytes ratio was significantly higher on 7 dpi (*P* < 0.05) than at the beginning of the experimental period (Figure 7) in the animals infected by strain ST121.

## Discussion

### Bacterial virulence genes

Differences were observed between strains ST121 and ST96. While isolate ST121 harboured all six egc cluster members, isolate ST96 lacked them. Although this gene cluster has been found in isolates of healthy individuals [38], egc-encoded proteins have been described to be involved in virulence [39–41], and the superantigenicity of egc-encoded proteins and rabbit toxicity has been confirmed [42]. The fact that these genes were detected in strain ST121 could be related to greater virulence in relation to strain ST96, and suggests that the egc cluster might be more important than other virulence determinants for staphylococcal rabbit pathogenesis. Furthermore, strain ST96 harboured *sak*, *scn*, *chp*, *selp* and phage integrase Sa3. The group of *S. aureus* Sa3int phages typically integrated into the *hlb* gene, which led to the negative conversion of β-haemolysin production [43]. These phages contain an immune evasion cluster (IEC) that encodes a combination of virulence factors [44]. In this case, strain ST96 carried a Sa3int phage, as well as *sak*, *scn*, *chp* and *selp*, and was negative to *hlb*, which could indicate that it is a phage integrated into the *hlb* gene with immune-evasion-cluster (IEC) type F [44], while isolate ST121 lacks IEC genes. These genes are typically detected in human strains, where they are grouped into the immune evasion cluster type. The strains from the same linage isolated from milkers or from cows with mastitis differ regarding the presence of the immune evasion cluster. This suggests that the lineage has adapted to the bovine host with loss of prophage [45]. Therefore, strain ST96 showed clear proximity with human strains, which rarely infect rabbits. Another difference was the detection of the *bbp* gene in only strain ST121, which could also contribute to the virulence of this strain. Vancraeynest et al. [46] detected only the adhesin Bbp in the isolates they previously characterised as high-virulence strains, which were not detected in other isolates. In human strains, *bbp* genes are associated with osteomyelitis and arthritis [47]. The profile of the bacterial virulence genes detected in strains ST121 and ST96 matched those previously described for clonal complexes CC121 and CC96 [29]. Although variation in molecular profiles has already been described depending on the genotype [14, 29, 48, 49], the detected genetic differences could justify variations in virulence between strain ST121 and strain ST96 as the gene expression and regulation of virulence elements in *S. aureus* isolates are generally controlled by global gene regulators [50]. Therefore, it would be necessary to increase knowledge about the gene expression and regulation of these virulence elements in strains ST121 and ST96 to better understand their role in the pathogenesis of staphylococcal infections.

### In vitro assays

To confirm the suspicion of previously observed genetic differences being responsible for a different virulence between both isolate types (ST121 and ST96), studies on haemolysis and cytotoxicity (PMN and macrophage lysis assays) were performed. Strain ST121 showed more cytotoxicity for erythrocytes, PMN and macrophages than strain ST96. These results would support the greater virulence of strains ST121 than strains ST96. Haemolysis (lysis of erythrocytes) is a significant virulence determinant of *S. aureus* and represents a crucial means for bacteria to acquire iron [14], whereas increased cytotoxicity in PMN and the macrophages of strain ST121 would affect two of the most important defence mechanisms of mammary glands against invading pathogens [51, 52].

### Experimental model

The genetic analysis and in vitro test results strongly suggest that both strain types (ST121 and ST96) differ in terms of their virulence. To clarify this point, experimental infection was carried out using very low infection doses (100 CFU) as a novelty to simulate natural infection conditions.

The infection with only 100 CFU was able to develop mastitis, a process characterised by the changes observed in the number of cell populations in both peripheral blood and mammary tissue. This experimental model clearly differed from most studies on *S. aureus*, where the inoculum usually oscillates, e.g.: 10^6^–10^9^ CFU [20, 25, 53, 54]. Inoculation of a few bacteria can better represent what actually happens in natural infection. This result confirmed that when the bacteria used for the experimental infection have naturally adapted to the host, high inocula concentrations are not necessary, which allows infection to develop similarly to how it occurs naturally by allowing bacteria a better chance to confront and challenge the host’s immune system [55].

#### Temperature evolution

Only three rabbits infected by strain ST121 had slight fever, and sporadically on 3 dpi, while no rabbit from the ST96 or control groups developed fever. This is a good indicator that the model does not generate excessive discomfort for animals and can be considered a relatively respectful model of animal welfare.

#### Macro- and microscopic lesions

Regarding the pathological findings, the lesions caused by strain ST121 were similar to those observed in chronic mastitis under natural conditions [10]. Conversely, the lesions caused by strain ST96 generated milder and more immature lesions. These findings can be interpreted as these bacteria not triggering an adequate inflammatory response by the host, which would result in milder immature lesions. Nevertheless, this reaction could still allow bacteria to colonise mammary tissue, cause mammary gland induration and severe infection. Indeed, under natural conditions, gross appearance is not enough to differentiate whether clinical mastitis was caused by strain ST96 or ST121. However, in the experimental infections, both strain types were not able to produce the same lesions in type and severity terms. Therefore, some circumstances that affect the host under natural conditions (e.g. prior immunosuppression) may be key for severe lesions to develop if ST96 infections appear.

#### Number of bacteria from lesions

The number of CFU was significantly bigger for the animals infected by ST121 than by ST96 (an increase of +97% CFU; *P* < 0.05). A large bacterial load has been described in natural mastitis cases caused by strains ST121 [10]. The bigger the bacterial load, the more likely bacteria disseminate to the environment, and suckling rabbits become infected by contaminated milk [6]. This reasoning could support the greater dissemination capacity of this strain.

#### Local immune response

Despite the difference noted in the macroscopical lesions developed by strains ST96 and ST121, very few differences were found in the counts of locally analysed leukocytes, except for the bigger number of macrophages in the nipples of the ST121-infected rabbit does. The fact that more macrophages were observed in the mammary glands infected by strains ST121 could indicate a more powerful inflammatory response than that generated by strains ST96. However, this response may prove inefficient as bacteria have been described to have mechanisms that support their destruction and evasion by these cells [56]. One report on lactating females indicates that the efficacy of phagocyte cells diminishes by the intake of fat vacuoles from milk [57]. Therefore, it would be interesting to perform phagocytic viability tests on these macrophages to clarify the role of these macrophages.

#### Peripheral immune response

The rabbits infected by strain ST121 had a higher proportion of circulating leukocytes than the ST96 group on 7 dpi and higher proportions of monocytes on 3 and 7 dpi versus 0 dpi. This increase in the monocytes counts in peripheral blood and macrophages in tissue observed in the animals infected by ST121 has also been reported in natural mastitis, for which a positive correlation has been described [11].

The rabbits infected by strain ST121 presented the lowest counts of total lymphocytes, B and CD5^+^, CD4^+^ and CD8^+^ lymphocytes on 1 and 3 dpi, and all the counts increased on 7 dpi. Therefore, most lymphocyte populations remained low until the end of infection, which directly affected their granulocytes/lymphocytes ratio because it was significantly higher on 7 dpi (*P* < 0.05) than at the beginning of the experimental period. The granulocytes/lymphocytes ratio has been reported as an immunological stress indicator, which is known to increase if diseases or infections are present [58].

Differences were detected in the number of circulating white blood cells according to strain *S. aureus* (ST96 or ST121) in the natural mastitis study. In the rabbits whose mammary glands were infected by strains of the ST96 lineage, relatively fewer granulocytes and more lymphocytes were detected in the blood samples compared to the strains of the ST121 lineage [11]. In this case, the same tendency was observed, but with no significant differences. This was probably due to the duration of infection, which was much more chronic for natural mastitis cases.

The results herein obtained highlight the differences in the pathogenicity of the employed strains, and suggest that the genetic differences detected between both strains are directly related to their pathogenicity. It has been described how rabbits can be infected by two types of *S. aureus* strains: high-virulence strains associated with a high mortality rate; low-virulence strains associated with sporadic infections and with slight economic loss [6]. In this work, ST96 could be considered a less virulent strain than ST121 based on their genetic differences, and on its behaviour after the in vitro and experimental experiments. As it is impossible to differentiate infections caused by both strain ST121 and ST96 under field conditions because they cause lesions of the same severity [11], other factors must be involved in infections developing under field conditions (immunosuppression of animals, stress, farm conditions, etc.) that favour the sudden appearance of aggressive outbreaks by low-virulent strains like ST96.

In conclusion, the devised infection model was adequate for evaluating differences in virulence between strains ST121 and ST96. Strain ST121 developed severer gross and histological mastitis than ST96 strain. The in vitro tests confirmed stronger virulence of strains ST121 vs. strains ST96, which implies greater cytotoxicity for erythrocytes, PMN and macrophages. The observed genetic differences could justify this stronger virulence: mainly strain ST121 harboured the egc cluster, while strain ST96 carried a Sa3int phage, probably integrated into the hlb gene with IEC type F.

## Supplementary information

**Additional file 1. Oligonucleotide primers for the amplification of genes** **, thermocycler programmes and references.**
