# Supplementary material for: Differences in virulence between the two more prevalent Staphylococcus aureus clonal complexes in rabbitries (CC121 and CC96) using an experimental model of mammary gland infection
Source: Vet Res. 2020 Feb 13;51:11. doi: 10.1186/s13567-020-0740-1 (PMC7020377; doi:10.1186/s13567-020-0740-1)
Supplement: Supplementary file 1 — Additional file 1. Oligonucleotide primers for the amplification of genes , thermocycler programmes and references. [file 13567_2020_740_MOESM1_ESM.docx]

**Additional file 1 Oligonucleotide primers for the amplification of genes, thermocycler programmes and references.**

| **Primer** | **Sequence (5’-3’)** | **Positive control** | **PCR^a^** | **Reference** |
| --- | --- | --- | --- | --- |
| *arcA*  arcA-1m  arcA-2c | GAGCCAGAAGTACGCGAG  CACGTAACTTGCTAGAACGAG | USA300 | 4 | [59] |
| *atlA*  atlA-1m  atlA-2c | AATGGTTGCATTAACGCTTGT  TTATCGTCGAACGATCATTAG | Newman | 4 | [15] |
| *bbp*  bbp-1m  bbp-2c | AACTACATCTAGTACTCAACAACAG  ATGTGCTTGAATAACACCATCATCT | RF122 | 2 | [29] |
| *clf*A  clfA-1m  clfA-2c | GTAGGTACGTTAATCGGTT  CTCATCAGGTTGTTCAGG | Newman | 1 | [29] |
| *clf*B  clfB-1m  clfB-2c | CAGCAGTAAATCCGAAAGACCC  CACCTTTAGGATTTGATGGTGC | Newman | 4 | [60] |
| *chp*  chp-1m  chp-2c | TTTACTTTTGAACCGTTTCCTAC  CGTCCTGAATTCTTAGTATGCATATTCATTAG | MSSA-476 | 2 | [61] |
| *cna*  cna-1m  cna-2c | AGTGGTTACTAATACTG  CAGGATAGATTGGTTTA | MSSA-476 | 2 | [29] |
| *ebp*S  ebpS-1m  ebpS-2c | CATCCAGAACCAATCGAAGAC  CTTAACAGTTACATCATCATGTTTATCTTTG | Newman | 2 | [29] |
| *eno*  eno-1m  eno-2c | ACGTGCAGCAGCTGACT  CAACAGCATYCTTCAGTACCTTC | Newman | 4 | [47] |
| *eta*  eta-1m  eta-2c | CTAGTGCATTTGTTATTCAA  TGCATTGACACCATAGTACT | 2020 | 4 | [29] |
| *etb*  etb-1m  etb-2c | ACGCGTATATACATTCAATT  TCCATCGATAATATACCTAA | 2020 | 4 | [29] |
| *fib*  fib-1m  fib-2c | GCGAAGGATACGGTCCAAGAGA  CAATTCGCTCTTGTAAGACCATT | Newman | 4 | [29] |
| *fmtB*  fmtB-1m  fmtB-2c | AATGAAGATGCGAATCATGTTG  CATCCATTTTTGTTTGCGTAGA | COL | 4 | [62] |
| *fnb*A  fnbA-1m  fnbA-2c | CACAACCAGCAAATATAG  CTGTGTGGTAATCAATGTC | 8325 | 2 | [29] |
| *fnb*B  fnbB-1m  fnbB-2c | GTAACAGCTAATGGTCGAATTGATACT  CAAGTTCGATAGGAGTACTATGTTC | 8325 | 4 | [29] |
| *hla*  hla-1m  hla-2c | CTTCAGGGTTTTCACCAGACTTCG  TTTCCAATTTGTTGAAGTCCAATGC | COL | 4 | [48] |
| *hlb*  hlb-1m  hlb-2c | GTTGGTGCTCTTACTGACAA  TGTGTACCGATAACGTGAAC | COL | 3 | [44] |
| *hld*  hld-1m  hld-2c | GAATTTGTTCACTGTGTCGATAATCC  AATTAAGGAAGGAGTGATTTCAATGG | USA300 | 6 | [48] |
| *hlg*  hlg-1m  hlg-2c | GCCAATCCGTTATTAGAAAATGC  CCATAGACGTAGCAACGGAT | Newman | 4 | [29] |
| *ica* A  icaA-1m  icaA-2c | CCAGAAAATTCCTCACCCGTATTAG  GTGTCTGACTTCGCTTTAATACAGCC | N315 | 1 | [29] |
| *integrase* Sa1  Sa1-1m  Sa1-2c | AAGCTAAGTTCGGGCACA  GTAATGTTTGGGAGCCAT | Mu50 | 4 | [43] |
| *integrase* Sa2  Sa2-1m  Sa2-2c | TCAAGTAACCCGTCAACTC  ATGTCTAAATGTGTGCGTG | N315 | 4 | [43] |
| *integrase* Sa3  Sa3-1m  Sa3-2c | GAAAAACAAACGGTGCTAT  TTATTGACTCTACAGGCTGA | USA300 | 4 | [43] |
| *integrase* Sa4  Sa4-1m  Sa4-2c | ATTGATATTAACGGAACTC TAAACTTATATGCGTGTGT | MSSA-476 | 4 | [43] |
| *integrase* Sa5  Sa5-1m  Sa5-2c | AAAGATGCCAAACTAGCTG CTTGTGGTTTTGTTCTGG | 8325 | 4 | [43] |
| *integrase* Sa6  Sa6-1m  Sa6-2c | GCCATCAATTCAAGGATAG TCTGCAGCTGAGGACAAT | Newman | 4 | [43] |
| *integrase* Sa7  Sa7-1m  Sa7-2c | GTCCGGTAGCTAGAGGTC GGCGTATGCTTGACTGTGT | Newman | 4 | [43] |
| *lukA,B*  lukAB-1m  lukAB-2c | GTGTTATTTGATTTCGTTCTATG  TTATTTCTTTTCATTATCATTAAGTACTT | Newman | 5 | [63] |
| *lukS,F-*PV  pvl-1m  pvl-2c | ATCATTAGGTAAAATGTCTGGACATGATCCA  GCATCAAGTGTATTGGATAGCAAAAGC | RN6390 | 4 | [29] |
| *lukM,F-*PV  lukMF-1m  lukMF-2c | TGGATGTTACCTATGCAACCTAC  GTTCGTTTCCATATAATGAATCACTAC | RF122 | 4 | [12] |
| *lukE,D*  lukED-1m  lukED-2c | TGAAAAAGGTTCAAAGTTGATACGAG  TGTATTCGATAGCAAAAGCAGTGCA | RF122 | 4 | [49] |
| *map/eap*  map/eap-1m  map/eap-2c | GCGAAATATACAGTTAATTT  ACTTTTTTAATGTCAGTTGC | Newman | 1 | [29] |
| *psmA*  psmA-1m  psmA-2c | CTTTCACATGGGTATCATTGCAGG  CAATAGCCATCGTTTTGTCCTCCT | RF122 | 6 | [48] |
| *psmB*  psmB-1m  psmB-2c | TAATAATGACGGCGCAAAATTAGG  GCAACGATGTCTACGATACTTGTGC | COL | 6 | [48] |
| *sak*  sak-1m  sak-2c | AAGGCGATGACGCGAGTTAT  GCGCTTGGATCTAATTCAAC | MSSA-476 | 2 | [61] |
| *scn*  scn-1m  scn-2c | AGCACAAGCTTGCCAACATCG  TTAATATTTACTTTTTAGTGC | MSSA-476 | 2 | [61] |
| *sdr*C  sdrC-1m  sdrC-2c | ACGACTATTAAACCAAGAAC  GTACTTGAAATAAGCGGTTG | Newman | 3 | [29] |
| *sdr*D  sdrD-1m  sdrD-2c | GGAAATAAAGTTGAAGTTTC  ACTTTGTCATCAACTGTAAT | Newman | 3 | [29] |
| *sdr*E  sdrE-1m  sdrE-2c | ATCAAGTACTCAAAAACAGC  TGGCTTGTTTCTTTACCTGC | Newman | 3 | [29] |
| *sea*  sea-1m  sea-2c | AAAGTCCCGATCAATTTATGGCTA  GTAATTAACCGAAGGTTCTGTAGA | MSSA-476 | 4 | [29] |
| *seb*  seb-1m  seb-2c | TCGCATCAAACTGACAAACG  GCAGGTACTCTATAAGTGCC | COL | 4 | [29] |
| *sec*  sec-1m  sec-2c | GACATAAAAGCTAGGAATTT  AAATCGGATTAACATTATCC | N315 | 4 | [29] |
| *sed*  sed-1m  sed-2c | CTAGTTTGGTAATATCTCCT  TAATGCTATATCTTATAGGG | FRI1151m | 4 | [29] |
| *see*  see-1m  see-2c | TAGATAAGGTTAAAACAAGC  TAACTTACCGTGGACCCTTC | FRI326 | 4 | [29] |
| *seg*  seg-1m  seg-2c | AATTATGTGAATGCTCAACCCGATC  AAACTTATATGGAACAAAAGGTACTAGTTC | N315 | 4 | [29] |
| *seh*  seh-1m  seh-2c | CAATCACATCATATGCGAAAGCAG  CATCTACCCAAACATTAGCACC | MSSA-476 | 4 | [29] |
| *sei*  sei-1m  sei-2c | CTCAAGGTGATATTGGTGTAGG  AAAAAACTTACAGGCAGTCCATCTC | N315 | 4 | [29] |
| *sej*  sej-1m  sej-2c | GGTTTTCAATGTTCTGGTGGT  AACCAACGGTTCTTTTGAGG | FRI1151m | 4 | [49] |
| *selk*  selk-1m  selk-2c | ATGGCGGAGTCACAGCTACT  TGCCGTTATGTCCATAAATGTT | COL | 4 | [29] |
| *sell*  sell-1m  sell-2c | CACCAGAATCACACCGCTTA  TCCCCTTATCAAAACCGCTAT | N315 | 4 | [29] |
| *selm*  selm-1m  selm-2c | CTATTAATCTTTGGGTTAATGGAGAAC  TTCAGTTTCGACAGTTTTGTTGTCAT | N315 | 4 | [29] |
| *seln*  seln-1m  seln-2c | ACGTGGCAATTAGACGAGTC  GATTGATCTTGATGATTATGAG | N315 | 4 | [29] |
| *selo*  selo-1m  selo-2c | GAGAGTTTGTGTAAGAAGTCAAGTG  GATTCTTTATGCTCCGAATGAGAA | N315 | 4 | [29] |
| *selp*  selp-1m  selp-2c | CTGAATTGCAGGGAACTGCT  ATTGGCGGTGTCTTTTGAAC | N315 | 4 | [29] |
| *selq*  selq-1m  selq-2c | GAACCTGAAAAGCTTCAAGGA  ATTCGCCAACGTAATTCCAC | N315 | 4 | [29] |
| *selu*  selu-1m  selu-2c | TAAAATAAATGGCTCTAAAATTGATGG  ATCCGCTGAAAAATAGCATTGAT | N315 | 4 | [29] |
| *tst*  tsst-1m  tsst-2c | CTAATCAAATAATCAAAACTGC  TTTCCAATAACCACCCGTTT | N315 | 3 | [29] |

^a^Thermocycler program : **1:** 94 °C for 3 min; 35 cycles of 94 °C for 30 s, 45 °C for 30 s, and 72 °C for 120 s; 72 °C for 5 min. **2:** 94 °C for 3 min; 35 cycles of 94 °C for 30 s, 50 °C for 30 s, and 72 °C for 120 s; 72 °C for 5 min. **3:** 94 °C for 3 min; 35 cycles of 94 °C for 30 s, 50 °C for 30 s, and 72 °C for 60 s; 72 °C for 5 min. **4:** 94 °C for 3 min; 35 cycles of 94 °C for 30 s, 55 °C for 30 s, and 72 °C for 60 s; 72 °C for 5 min. **5:** 94 °C for 3 min; 35 cycles of 94 °C for 30 s, 55 °C for 30 s, and 72 °C for 120 s; 72 °C for 10 min. **6:** 94 ºC for 5 min; 30 cycles of 94 °C for 30 s; 58 °C for 20 s, and 72 °C for 20 s.; and 72 °C for 5 min.
